# Application of the Willis Covered Stent in the Treatment of Blood Blister-Like Aneurysms: A Single-Center Experience

**DOI:** 10.3389/fneur.2022.882880

**Published:** 2022-05-18

**Authors:** Wei Fang, Jia Yu, Yufeng Liu, Peng Sun, Zijian Yang, Zhenwei Zhao, Yue He, Jianping Deng, Tao Zhang

**Affiliations:** ^1^Department of Neurosurgery, Tangdu Hospital, Air Force Medical University, Xi'an, China; ^2^Tongji Hospital, Tongji Medical College, Huazhong University of Science and Technology, Wuhan, China

**Keywords:** blood blister-like aneurysm, endovascular, covered stent, internal carotid artery, subarachnoid hemorrhage

## Abstract

**Objective:**

To evaluate the effectiveness of the Willis covered stent (WCS) in the treatment of ruptured blood blister-like aneurysms (BBAs) of the internal carotid artery (ICA).

**Method:**

The clinical data of 16 patients consecutively treated with WCSs from December 2015 to January 2019 were retrospectively analyzed. Clinical data and angiographic findings were analyzed by two experienced neuroradiologists and neurosurgeons, including age, sex, Hunt and Hess (H&H) grade at admission, modified Rankin scale (mRS) score, aneurysm size, and location, the diameter of the patent artery in proximal and distal ends, stent size, rate of aneurysm occlusion, procedure-related complications, and follow-up.

**Results:**

All the 16 patients (five males, 11 females) with ICA BBAs underwent WCS deployment successfully. The median age was 49 years (range, 29–72). All patients had complete aneurysm occlusion on immediate postoperative angiography. Anterior choroidal artery (AChA) was occluded in one patient accidentally while no obvious neurological dysfunction was observed. However, this patient underwent subarachnoid hemorrhage 1 day after the treatment; endoleak and aneurysm recurred, and the patient died 10 days later. Therefore, the effective rate of WCS treatment was 93.8% (15/16), and procedure-related complications rate was 6.3% (1/16). Moreover, one patient was urgently treated because of accidental aneurysm rupture after anesthesia, and external ventricular drainage was then performed postoperatively. Another patient developed coma and hemiplegia 3 days after treatment, with emergency angiography showing in-stent thrombosis and ICA occlusion which was recanalized with arterial rt-PA thrombolysis; the patient recovered completely. The clinical follow-up period was 3–30 months in 14 patients. The mRS scores were 0 in 12 patients (85.7%) and 4 in 1 case (7.1%), while 1 patient (7.1%) died 6 months postoperatively for unknown reasons. Angiographic follow-up was performed in 13 patients, and no recurrence was observed. However, ICA occlusion without neurological deficit was observed in one patient.

**Conclusion:**

Based on careful preoperative evaluation, appropriate WCS size selection, and precise surgical operation, WCSs may provide an alternative and effective solution for blood BBAs *via* aneurysm isolation and ICA reconstruction immediately; However, further follow-up studies with larger samples are required.

## Introduction

Blood blister-like aneurysm (BBA) is often characterized with a fragile wall, a wide neck, and lack of distinguishable normal boundary between the aneurysm neck and parent vessel (1). Due to its special pathological features, neurosurgical treatment for BBA has been a big challenge compounded with the high odds of intraoperative rupture and postoperative recurrence. Different treatment modalities, including direct clipping, suturing, wrapping, coiling, stent-assisted coiling, multiple overlapping stents, and flow–diverter, have been applied for this terrible disease. Endovascular treatments have a lower risk of intraprocedural rupture but lower rates of aneurysm obliteration, while microsurgical treatments have higher rates of aneurysm obliteration but higher risk of intraprocedural rupture and subsequent ICA sacrifice (2–5). Among all these treatments, the most optimal management option for BBAs remains uncertain (1, 3–6).

In recent years, Willis covered stents (WCSs) have been specially designed for intracranial application, which brings a new option for the treatment of aneurysms, especially for BBAs (7). The WCS deployment has unique advantages compared with other treatments. It can isolate aneurysm and reconstruct the parent artery instantly without manipulations in the aneurysm (8). Several retrospective studies have evaluated the effectiveness of WCSs for BBAs with promising results (3, 9, 10). However, whether it is a reasonable choice for the BBA remains debatable considering the technical details. Here, we report our experience and evaluate the effectiveness in the treatment of BBAs of the internal carotid artery (ICA) with the WCS in 16 patients.

## Materials and Methods

### Patients

A retrospective study was performed in 16 patients with ruptured ICA BBAs treated with WCSs from December 2015 to January 2019. The examined data included patient's age, sex, Hunt and Hess (H&H) grade at admission, modified Rankin scale (mRS) score, aneurysm size and location, the diameter of the patent artery in proximal and distal ends, stent size, rate of aneurysm occlusion, procedure-related complications, and follow-up. This study was approved by the Ethics Committee of Tandu Hospital. Written informed consent was obtained from each patient for the purpose of research.

### The CT and DSA Diagnostic Criteria of BBA

All patients were examined clinically and radiologically by cerebral computed tomography (CT) to confirm subarachnoid hemorrhage before treatment. Aneurysms arising from the anterior wall of the ICA may be saccular aneurysms or blister-like aneurysms. However, these two subgroups are difficult to be identified angiographically. The most reliable method to diagnose BBA is microsurgical morphology and pathology. As the technical limitation of endovascular therapy, we adopt six criteria to diagnose BBA according to the following literature: (1) Aneurysms located at the non-branching sites of the supraclinoid ICA. (2) Typical aneurysm features, such as a wide neck and a typical blister-like shape. (3) Initially small size (maximum diameter <10 mm). (4) Ruptured, presented with SAH. (5) Rapid growth (<2 weeks) on repeated angiograms. (6) Irregular wall of the aneurysm or the parent artery. An aneurysm was diagnosed as BBA when criteria 1–4 were fulfilled and either of criteria 5 or 6 was matched (2, 11–13).

### WCS and Stent Deployment Procedure

The WCS consists of three parts, including a bared balloon-expandable stent, an expandable polytetrafluoroethylene (ePTFE) membrane, and a low-pressure flexible balloon catheter. Various diameters (3.5, 4.0, and 4.5 mm) and different lengths (7, 10, 13, and 16 mm) have been designed for different conditions. The general principle for stent selection is that the length of the stent must exceed the aneurysm neck at least by 2 mm for both sides, with the diameter of the stent imperatively exceeding the parent artery by 0–0.5 mm (3, 9).

Endovascular treatment was performed under general anesthesia and systemic venous heparinization. An 8F guide catheter (Envoy, Codman Neurovascular, USA) was placed into the common carotid artery (CCA) or the initial part of the ICA. A 5F Navien (ev3/Covidien, USA) supporting the catheter was generally advanced to the M1 segment of the middle cerebral artery (MCA) with an XT-27 microcatheter (Stryker, USA) and a 0.014'-microguidewire (Sychro, Stryker, USA) under roadmap guidelines. In case of a too tortuous proximal vessel, the 5F Navien was then advanced to the C4 segment of the ICA at least. With a microguidewire placed on the M2 segment of the MCA, the WCS was then carefully delivered upward to the target location. After angiographic confirmation was made that the stent had completely covered the neck of the aneurysm and not covering any important perforators, including the ophthalmic (OA) and anterior choroidal arteries, the stent was then deployed by balloon inflation at 5–6 atm. In case of an endoleak, the balloon was re-inflated with higher pressure until it was eliminated. However, the manipulation for re-inflation would not be performed for more than 3 times. If the endoleak persisted, another WCS could be deployed. The postoperative CT and neurologic examination were performed routinely to rule out intracranial hemorrhage or ischemic events.

### Antithrombotic Treatment

Intravenous tirofiban hydrochloride was administered immediately after stent deployment at an initial bolus of 10 μg/kg within 3 min and maintained at a rate of 0.15 μg/kg/min for 36 h. A loading dose of 300-mg or 75-mg clopidogrel and 300-mg or 100-mg aspirin was administered 6 h before tirofiban withdrawal. All patients were maintained on dual antiplatelet therapy (75-mg/day clopidogrel and 100-mg/day aspirin) for a minimum of 6 months. Aspirin (100 mg/day) was then taken for at least 1 year. Before discharge, all patients underwent thrombosis elastography (TEG) to confirm the drug effect.

### Follow-Up and Postoperative Outcome Evaluation

Among all 16 patients, 14 were assessed by angiography 7 days post-treatment. The other 2 patients received emergency angiography 1- and 3-days post-treatment, respectively, for deteriorative neurological symptoms. All patients were then followed up at 1, 3, 6, and 12 months, *via* clinical and angiographic assessments.

## Results

### General Characteristics

Our cohort consisted of 16 patients (11 women and 5 men) with a median age of 49 years (range, 29–72). As shown in Table 1, patients with primary H&H grade II accounted for 56.25%, and the BBAs were mainly located at the ICA C6 segment. One patient received decompressive craniectomy before admission to our hospital. One patient received an additional WCS after primary stent deployment for continuous endoleak.

**Table 1:** Demographic, clinical, CT data, endovascular treatment, and follow-up outcomes of the 16 patients.

**Patient no**.	**Sex/age** **(years)**	**H&H grade**	**mRS Score**	**Aneurysm location**	**Previous treatment**	**Aneurysm size**	**Diameter of the patent artery**	**Stent size (mm)**	**Immediate results**	**Complications**	**mRS in discharge**	**mRS score at final clinical follow-up (months)**	**Aneurysm occlusion**	**In stent stenosis**
1	M/45	I	1	L-ICA C6	NA	1.1^*^2.6	4.0/3.8	4.0 × 10	Complete occlusion	NA	1	0 (12)	Complete	NA
2	F/72	I	0	R-ICA C5	NA	2.3^*^2.9	3.3/2.9	3.5 × 7	Complete occlusion	NA	0	Lost	Lost	Lost
3	F/43	I	1	L-ICA C6	NA	3.8^*^6.9	3.2/2.9	3.5 × 10	Complete occlusion	Ruptured during preoperative angiography	1	0 (12)	Complete	stenosis
4	F/50	II	1	R-ICA C6	NA	3.0^*^1.5	3.4/3.2	3.5 × 7	Complete occlusion	NA	1	0 (30)	Complete	NA
5	F/43	II	1	L-ICA C5	NA	3.1^*^2.4	3.8/3.5	4.0 × 10	Complete occlusion	NA	1	0 (10)	Complete	NA
6	F/59	I	1	L-ICA C6	NA	1.0^*^1.2	3.4/3.1	3.5 × 10	Complete occlusion	In-stent stenosis	1	Dead (6)	NA	Dead
7	F/49	II	1	R-ICA C6	NA	5.1^*^2.5	3.1/2.8	3.5 × 7	Complete occlusion	NA	1	0 (9)	Complete	NA
8	M/65	II	1	L-ICA C5	NA	2.2^*^1.9	3.5/3.2	3.5 × 10&3.5 × 7	Complete occlusion	NA	1	0 (3)	Complete	NA
9	F/40	II	1	L-ICA C6	NA	6.0^*^3.5	4.0/3.9	4.0 × 7	Complete occlusion	NA	1	0 (7)	Complete	NA
10	M/59	II	1	R-ICA C6	NA	6.7^*^3.0	3.1/3.0	3.5 × 7	Complete occlusion	NA	1	0 (12)	Complete	NA
11	F/50	II	2	R-ICA C6	NA	4.0^*^1.9	3.5/3.5	3.5 × 7	Complete occlusion	NA	2	0 (14)	Complete	NA
12	F/29	II	1	R-ICA C6	NA	1.2^*^2.7	3.6/3.4	4.0 × 7	Complete occlusion	NA	1	0 (10)	Complete	NA
13	M/49	III	2	L-ICA C7	NA	1.8^*^5.1	3.5/3.3	3.5 × 10	Complete occlusion	L-AChA occlusion and aneurysm recurrence	Dead	NA	NA	Dead
14	F/47	II	1	R-ICA C7	NA	3.6^*^2.1	3.4/2.6	3.5 × 7	Complete occlusion	NA	1	0 (9)	Complete	NA
15	F/61	IV	5	R-ICA C6	Decompressive craniectomy	6.5^*^2.9	3.3/2.8	3.5 × 7	Complete occlusion	NA	4	4 (18)	Complete	NA
16	M/49	I	1	L-ICA C6	NA	1.5^*^2.4	3.2/3.1	3.5 × 7	Complete occlusion	NA	1	0 (9)	Complete	NA

*Patient No., patient number; H&H, Hunt and Hess; mRS, modified Rankin scale; L-ICA, left internal carotid artery; R-ICA, right internal carotid artery; NA, not applicable; M, male; F, female; AHCA, Anterior choroidal artery*.

### Procedural Results and Complications

A total of 17 stents were successfully deployed and all 16 aneurysms were completely occluded for immediate angiography postoperatively. In our definition, procedure-related complication only means complications that occurred during stent delivery and deployment procedure. Thus, the procedure-related complications occurred in one case (6.3%). In this patient, the AChA was covered by the WCS accidentally and was subsequently occluded (see Patient No. 13 in Table 1) during immediate angiography, while no newly neurological deficit occurred postoperatively. Three patients experienced perioperative complications. The patient with AChA occlusion fell into coma on the next day, and CT scan showed a significant increase in cerebral hemorrhage. Previously occluded BBA and AChA were visualized again in subsequent angiography, the reason for which was considered to be the shrinkage of the WCS membrane. Although we advised performing a craniotomy to save his life, the patient's family refused any further treatment. This patient died 10 days later. Moreover, one patient (Patient No. 3 in Table 1) suffered an unexpected aneurysm rupture during the roadmap procedure after anesthesia. After an urgent WCS deployment, the aneurysm was completely occluded, and lateral ventricle external drainage was performed immediately post-treatment. No neurological deficit occurred postoperatively and the mRS score was 1 at discharge. However, ipsilateral ICA occlusion was found in a follow-up Magnetic Resonance Angiography (MRA) 12 months postoperatively in this patient. In another case, in-stent thrombosis was found by angiography 2 days after the treatment (Patient No. 6 in Table 1). However, revascularization was achieved by intra-arterial administration of 3.5 mg of tirofiban and 9 mg of alteplase, and the patient had no neurological deficit at discharge.

### Follow-Up Results

As described in Table 1, 14 patients received clinical and radiographic evaluations at follow-up ranging from 3 to 30 months, and 12 (85.7%) obtained good outcomes (mRS score = 0). One case (7.1%) improved from the mRS score of 5 to 4. Another patient died 6 months after treatment for an unknown reason. Of all 16 patients, 13 (81.25%) achieved good recovery after treatment and one was lost to follow-up.

Angiographic follow-up was performed in 13 patients from 3 to 14 months after the treatment. No aneurysm recurrence was found, but ipsilateral ICA occlusion was found in case 2 (**Figure 2**).

### Special Cases

#### Patient 1

**Figure 1:**
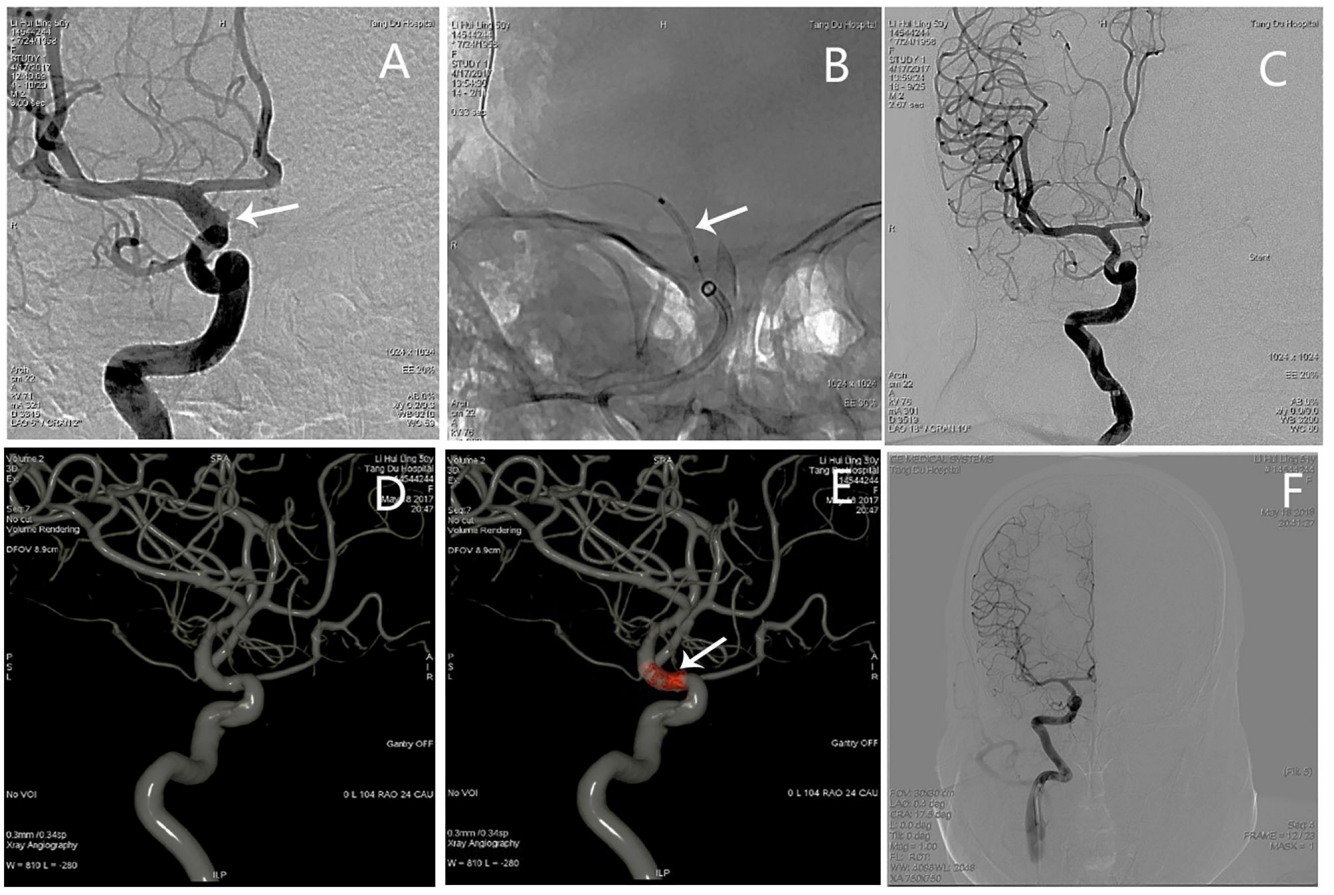
Angiographic images of a 50-year-old woman (see Patient No. 4 in Table 1) with a right ICA C6 BBA. **(A)** A BBA (3.0*1.5 mm) located on the anterior wall of the C6 segment. **(B)** A 3.5*7-mm WCS was deployed (arrow shows). **(C)** Immediate postoperative angiography showed complete aneurysm occlusion, and the OA and ACHA were not affected. **(D)** The 3D-DSA showing complete aneurysm occlusion and the parent artery's morphology. **(E)** Dual volume technology showing the morphology of the stent and its relationship with the parent artery. **(F)** Angiography images showing complete aneurysm occlusion at the 13-month follow-up examination.

An adult patient (see Patient No. 4 in Table 1) was admitted to our hospital with intense headache (Figure 1). Emergency angiography demonstrated a BBA (3.0^*^1.5 mm) located on the anterior wall of the C6 segment of the R-ICA; the diameters of the patent artery in proximal and distal ends were 3.4 and 3.2 mm, respectively. A 3.5^*^7-mm WCS was deployed successfully, and immediate postoperative angiography showed complete aneurysm occlusion. The 13-month follow-up angiography showed complete occlusion with good parent artery patency. The patient had a satisfactory clinical status with the mRS score of 0 at the last follow-up examination (Figure 1).

#### Patient 2

**Figure 2:**
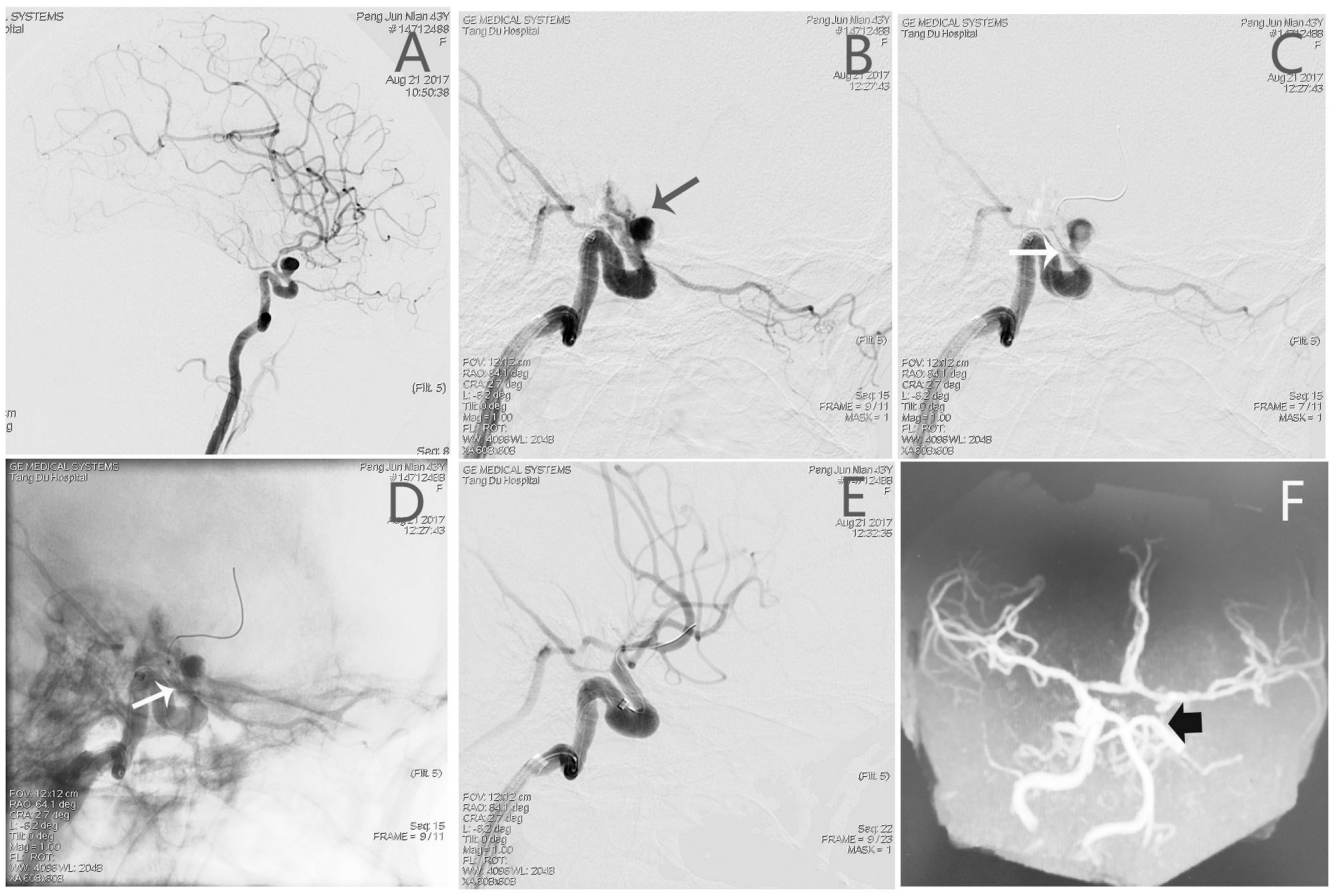
Angiographic images of a 43-year-old woman (see Patient No. 3 in Table 1) with a L-ICA C6 BBA. **(A)** A BBA (3.8*6.9 mm) located on the anterior wall of the C6 segment. **(B)** The aneurysm ruptured during preoperative angiography (black arrow). **(C,D)** A 3.5*10-mm WCS deployed immediately (white arrow). **(E)** Postoperative angiography showing complete aneurysm occlusion, and the OA was also affected. **(F)** The MRA images showing the L-ICA was occluded completely.

An adult patient (see Patient No. 3 in Table 1) was admitted to our hospital with intense headache for 3 days (Figure 2). The aneurysm ruptured during preoperative angiography unexpectedly. A BBA (3.8^*^6.9 mm) located on the anterior wall of the C6 segment of the R-ICA; the diameters of the patent artery in proximal and distal ends were 3.2 and 2.9 mm, respectively. After a 3.5^*^10 mm WCS was deployed immediately, the aneurysm was completely occluded, and the lateral ventricle external drainage was performed. Although the mRS score was 0 at 12 months follow-up, ipsilateral ICA occlusion was found by an MRA examination (Figure 2).

#### Patient 3

**Figure 3:**
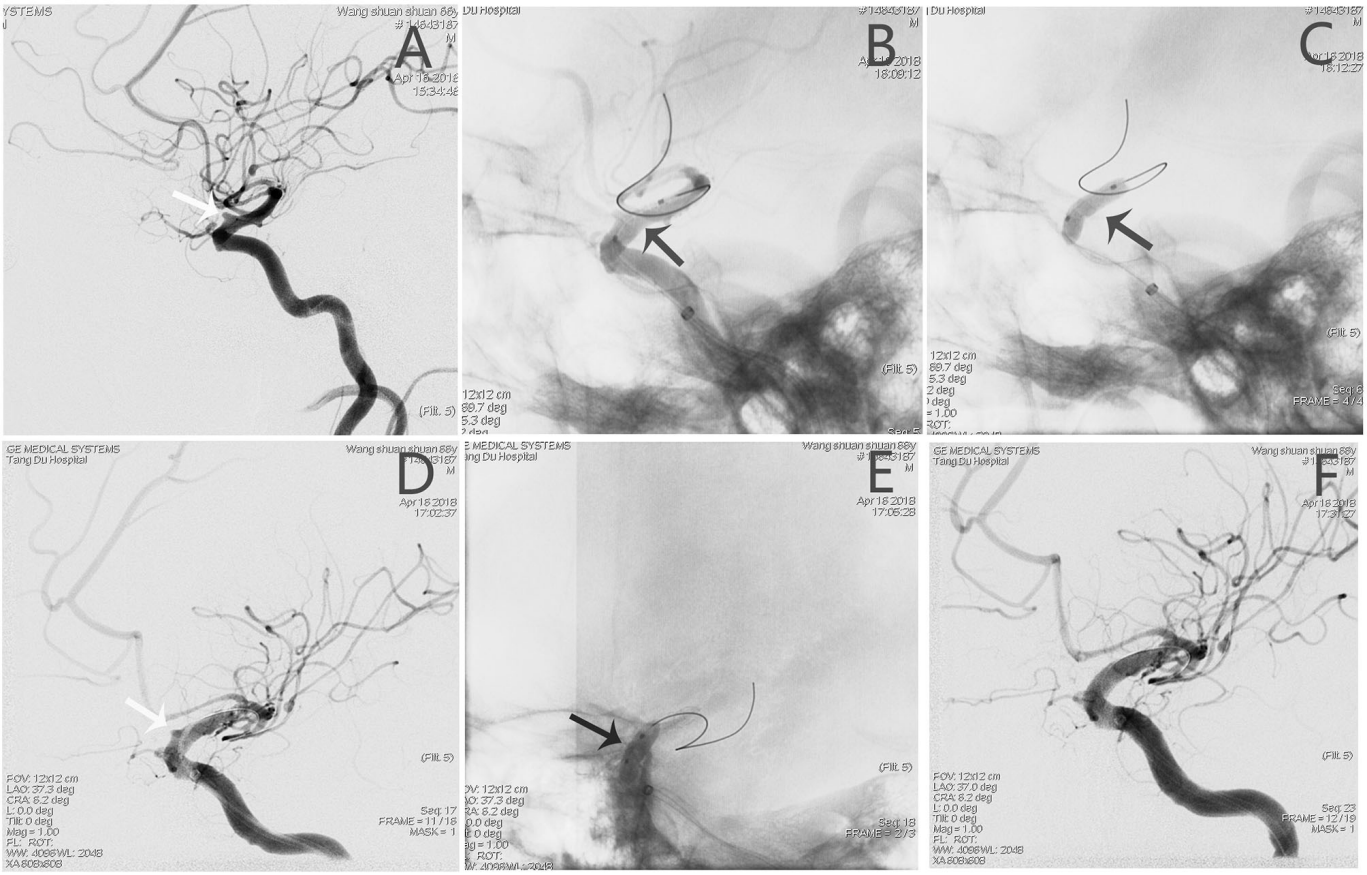
Angiographic images of a 65-year-old man (see Patient No. 8 in Table 1) with a L-ICA C6 BBA. **(A)** A BBA (2.2*1.9 mm) located on the anterior wall of the C6 segment (white arrow). **(B,C)** A 3.5*10-mm WCS was deployed (black arrow). **(D)** After re-inflation, the endoleak phenomenon remained (white arrow). **(E)** A 3.5*7-mm WCS was joined proximally. **(F)** Angiographic images showing complete aneurysm occlusion.

An adult patient (see Patient No. 8 in Table 1) was admitted to our hospital with intense headache and a history of vomiting for 6 h (Figure 3). The BBA (2.2^*^1.9 mm) was located on the anterior wall of the C6 segment of the left internal carotid artery (L-ICA); the diameters of the patent artery in proximal and distal ends were 3.5 and 2.2 mm, respectively. After a 3.5^*^10-mm WCS deployment and two balloon re-inflation procedures, the endoleak phenomenon remained. Therefore, another 3.5^*^7 mm WCS was joined proximally, and the aneurysm was completely occluded (Figure 3).

#### Patient 4

**Figure 4:**
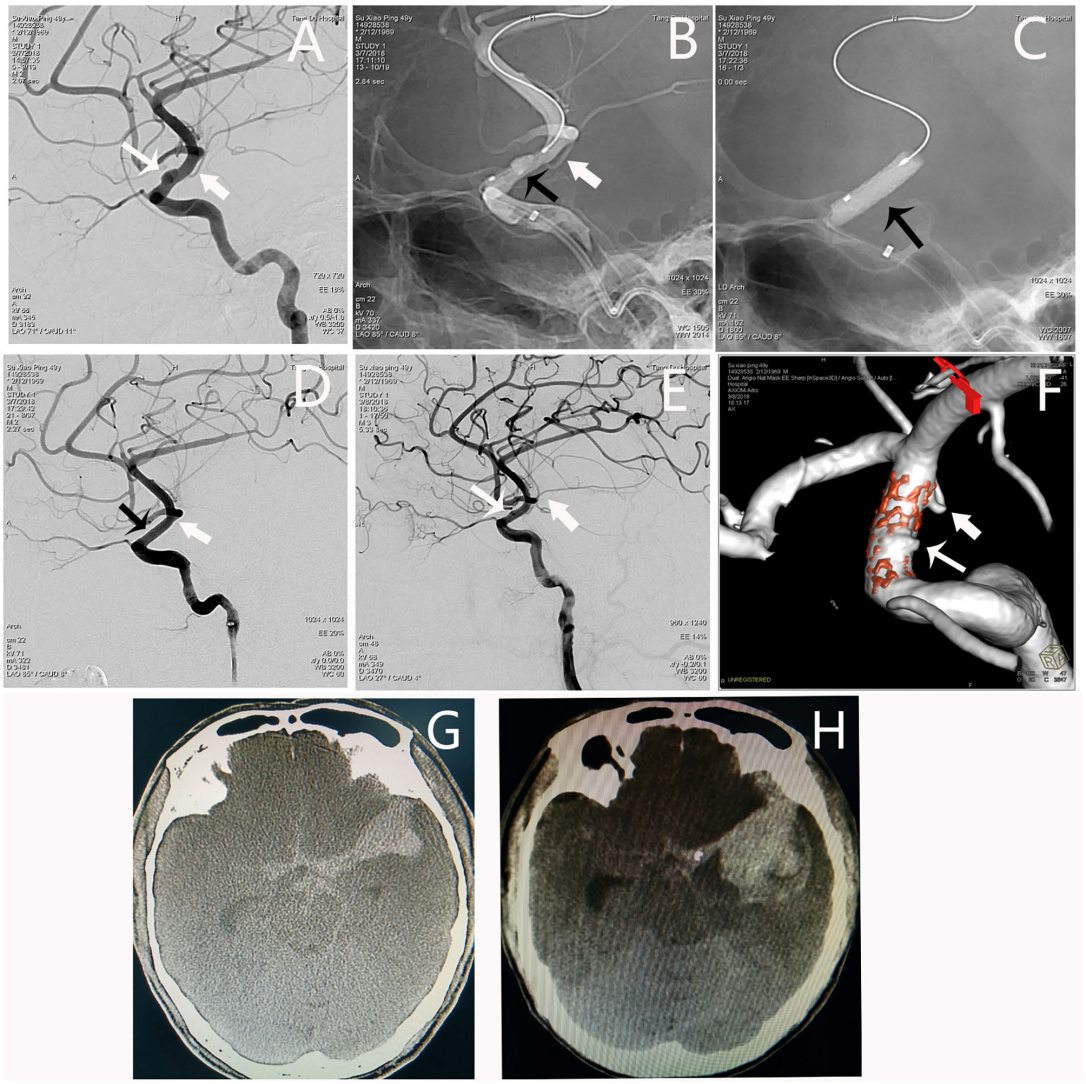
Angiographic images of a 49-year-old man (Patient no. 13 in Table 1) with a L-ICA C6 BBA. **(A)** A BBA (1.8*5.1 mm) located on the lateral wall of the C6 segment (white thin arrow); the AHCA was indicated by the white fat arrow. **(B,C)** A 3.5*10-mm WCS was deployed (black arrow). **(D)** Immediate postoperative angiography showing the aneurysm and the ACHA were both occluded. **(E,F)** DSA and dual volume technology showing aneurysm recurrence, and the AChA appeared again. **(G)** Preoperative CT scan showing subarachnoid hemorrhage. **(H)** The CT scan showing significantly increased hematoma as the patient fell in coma.

An adult patient (see Patient No. 13 in Table 1) was admitted to our hospital with intense headache for 1 day. The BBA (1.8^*^5.1 mm) was located on the lateral wall of the C6 segment of the L-ICA; the diameters of the patent artery in proximal and distal ends were 3.5 and 3.3 mm, respectively. After a 3.5^*^10-mm WCS deployment, the aneurysm and the AChA were both occluded. The occlusion of the AChA was an accident that was not part of our surgical plan, as we had already considered avoiding this vessel when selecting the stent length and designing the stent deployment. However, the stent migration during balloon dilation and occlude the AChA. Although the patient had no new neurological deficit postoperatively, he fell into coma on the second day. Emergency angiography showed aneurysm recurrence, and the AChA was visualized again (Figure 4).

## Discussion

Although neurosurgical techniques and instruments have made great progress in the past decades. Both treatments for BBAs are still greatly challenging for neurosurgeons and neuroradiologists due to the special features of BBAs. Currently, the main treatment options for BBAs include surgical clipping, artery suturing, wrapping and clipping, stent-assisted coiling embolization, multi-stent implantation, and flow-diverting stents (2, 6, 14–17). However, a rescue or second treatment was required in 21% patients, and the overall morbidity and mortality rates were 17 and 15%, respectively. As endovascular treatment offers a lower procedural-related complication rate compared with surgical approaches, interventional therapy appears to be a more optimal treatment option for BBAs, with a more favorable patient outcome (16, 18). It should be pointed out that flow–diverter stent application in BBAs has achieved relatively more satisfactory results compared with traditional endovascular treatment in recent years (19–22). However, the lower rate of the immediate aneurysm occlusion and the use of antiplatelet agents make it controversial.

Since Li et al. first reported the treatment of intracranial pseudoaneurysms with a Willis coated stent in 2006 (7), more and more studies have reported the efficacy of WCSs in the treatment of BBAs, with the majority of patients having promising results (3, 9, 10, 23). The prominent advantage of WCS deployment is the immediate occlusion of aneurysms from the circulation, especially compared with the flow–diverter stents. Furthermore, the deployment of the WCS involves no manipulations focusing on the aneurysm cavity, thus reducing the risk of procedural-related rupture. As described here, all the 16 patients achieved immediate aneurysm occlusion after stent deployment, and no procedural-related rupture occurred. Compared with surgical procedures and other endovascular treatments, WCS deployment is technically simple and safe.

Although WCSs have many advantages, their limitations should also be considered. As WCS is stiffer than the ordinary stents due to its membrane characteristics and the microguidewire transportation system, its delivery is much more difficult than for another stent, especially during tortuous vascular access (24). Therefore, this stent is not recommended for the tortuous vascular approach. According to our experience, the combined use of an 8F guiding catheter and a 5F Navien support catheter is an optional choice. In most cases, we preferred to advance the 5F Navien beyond the aneurysmal neck to the M1 segment of the MCA; thus, WCSs could be delivered within the Navien to the target position. In some cases, with tortuous proximal vessels, a 5F Navien would be advanced to the C4 segment of the ICA for best support. Moreover, vasospasm, calcification, and stenosis should also be considered cautiously as they may increase the procedure risks. Vasospasm may influence the accurate selection for stent size while inappropriate stent may lead to poor stent apposition and aneurysm recurrence. Calcification and stenosis may influence stent delivery, balloon inflation, and stent apposition. As for the choice of stent length, branching and perforating artery occlusion is a major concern. Therefore, the appropriate size and deployment precisely of the cover stent is the key for the operation. For the clinoidal segment of the ICA, the OA, posterior communicating (PComA), and AChA are the three major arteries close to the BBA site. During WCS application, we tried our best not to sacrifice these arteries by choosing appropriate stents. The PComA was the primary concern in this study; if the PComA is absent or hypoplastic, it could be sacrificed without hesitation. As for the OA, because the deep and superficial anastomotic network will ensure the blood supply of the central retina artery, only a few patients experience blindness when OA is occluded (3, 25). The AChA provides the blood supply to many vital structures, including the posterior two-thirds of the internal capsule, adjacent optic nerve and auditory radiation, the medial portion of the globus pallidus, and the tail of the caudate nucleus (3, 26, 27). Acute occlusion of the AChA would result in catastrophic consequences that could not be predicted. In this study, the AChA was occluded in one patient owing to stent migration during balloon dilation. Although this patient had no neurological deficit after the treatment, he died several days later because of BBA recurrence and rebleeding.

As for the choice of stent diameter, endoleak is another major concern, although it is more likely to happen in an application for giant aneurysms (8, 10, 28). Endoleak issues may include stent shrinkage, inappropriate choice of stent, and irregular appearance of the parent artery. Based on our experience in preventing endoleak, a stent equal to or a little bigger than the artery in diameter is preferred. In this study, immediate endoleak was observed in two cases which were solved by balloon re-inflation with higher pressure in one case and deployment of a second stent proximally in another case. The significance of the early angiographic review should also be emphasized for WCS application to prevent BBA recurrence. In this study, one patient (see Patient No. 13 in Table 1) had aneurysm recurrence and serious re-hemorrhage the next day post-treatment, which was confirmed by angiographic review. Stent shrinkage caused by inappropriate selection of stent diameter might be the reason. Another possible reason may be the vasospasm caused by subarachnoid hemorrhage. The diameter of the parent artery may be not accurate. Therefore, when the vasospasm is relieved, the increase in the artery diameter may cause poor stent apposition. To our knowledge, the stent should be equal to or slightly larger than the target vessel in diameter. If the stent applied is smaller than the target vessel (although the balloon dilated with higher pressure), the stent might retract to its normal size and the covered membrane could not isolate the lesion, which would result in aneurysm recurrence and re-bleeding. Furthermore, the vessel spasm should also be cautiously considered in the measurement of vessels. As ipsilateral vessel spasm might be serious, we suggest referring to a contralateral vessel in diameter during measurement.

In this study, in-stent stenosis was found in one patient (see Patient No. 3 in Table 1) during the angiographic follow-up 6 months later. The main reason is that WCSs are more thrombogenic than the ordinary stents. Stent malposition may also increase the risk of in-stent thrombosis.

Although this study showed a promising result for WCS application in BBA treatment, there were still two major limitations. First, the long-term follow-up should be carried out to confirm the safety and efficacy of WCSs in BBA. Second, the number of patients was relatively small, and larger clinical trials are needed to confirm these results.

## Conclusion

The WCSs may provide an alternative and effective solution for BBAs by aneurysm isolation and immediate ICA reconstruction. However, further follow-up studies with larger samples are required for confirmation.

## Data Availability Statement

The original contributions presented in the study are included in the article/supplementary material, further inquiries can be directed to the corresponding author/s.

## Ethics Statement

The studies involving human participants were reviewed and approved by Institutional Review Board, Tang DU Hospital. The patients/participants provided their written informed consent to participate in this study. Written informed consent was obtained from the individual(s) for the publication of any potentially identifiable images or data included in this article.

## Author Contributions

WF acquired and analyzed data, and wrote the first draft of the manuscript. JY, YL, and PS performed imaging analyses and acquired related data. ZY acquired clinical data. ZZ and YH contributed to the database. JD and TZ contributed to the conception and design of the study and reviewed the manuscript. All authors contributed to manuscript revision, read, and approved the submitted version.

## Conflict of Interest

The authors declare that the research was conducted in the absence of any commercial or financial relationships that could be construed as a potential conflict of interest.

## Publisher's Note

All claims expressed in this article are solely those of the authors and do not necessarily represent those of their affiliated organizations, or those of the publisher, the editors and the reviewers. Any product that may be evaluated in this article, or claim that may be made by its manufacturer, is not guaranteed or endorsed by the publisher.
